# Prevalence of pre-diabetes and risk factors among secondary school adolescents in Osogbo Local Government Area, Osun State, Nigeria

**DOI:** 10.4314/ahs.v21i3.41

**Published:** 2021-09

**Authors:** Nafisat O Akintayo-Usman, Funmilayo A Okanlawon, Saheed O Usman

**Affiliations:** 1 Nurse Tutors' Programme, University College Hospital, Ibadan, Nigeria; 2 Department of Nursing, College of Medicine, University of Ibadan, Nigeria; 3 Department of Chemical Pathology, Nnamdi Azikiwe University Awka, Nigeria

**Keywords:** Pre-diabetes, adolescents, individual factors, interpersonal factors, community factors

## Abstract

**Background:**

Pre-diabetes is an emerging public health challenge in sub-Saharan Africa.

**Objectives:**

To estimate prevalence of pre-diabetes and assess its associated factors among adolescents. The risk factors were divided into individual, interpersonal and community factors, adapting socio-ecological model.

**Methods:**

This study utilised a cross-sectional descriptive survey. The target population was secondary school adolescents of Osogbo Local Government. Questionnaire was used to interview 405 participants through multi-stage sampling. Prediabetes was measure through fasting blood glucose.

**Results:**

Findings revealed prevalence rate of 9.4%. Individual factors identified to be significant include age, religion and family history. Further analysis showed adolescents with normal BMI and high BP are likely to develop pre-diabetes when compared to those with underweight and normal BP respectively. Among interpersonal factors, parents' dietary habit was significant. Also, adolescents with employed parents were likely to develop pre-diabetes compared to those with unemployed parents. Lastly, availability of healthy food in school was the only statistically significant community factor. Hence, the more availability of food, fruits and vegetables in schools, the less likelihood of developing prediabetes.

**Conclusion:**

These findings affirmed that prediabetes is becoming common problem among Nigerian adolescents. There is therefore need for stakeholders to face this challenge before it becomes endemic.

## Introduction

More African people will die from non-communicable diseases (NCDs) than infectious diseases by 2030; with 27% increase in deaths expected over next ten years^1^,^2^. Meanwhile 75% of 415 million people living with diabetes are from low- and middle-income countries, with a prediction of reaching 642 million by 2040 3. Prediabetes and Diabetes Mellitus (DM) are increasing problems in sub-Saharan Africa with type II DM being the most common^4^.

Prediabetes is defined as plasma glucose levels that are elevated above the normal range, but below the threshold for diabetes. World Health Organisation (WHO) defines prediabetes as Fasting Plasma Glucose, FPG level of 110 – 125 mg/dL and/or Oral Glucose Tolerance Test, OGTT of 140 – 200 mg/dl; while International Society for Pediatric Adolescent Diabetes (ISPAD) defines it as FPG of 100 – 125 mg/dl or OGTT of 140 – 200 mg/dl 5,6. It does not have to result in diabetes if identified early, as lifestyle changes are enough to prevent it from progressing to diabetes. It is therefore important for adolescents to know their pre-diabetic status, because research has shown that some long-term complications associated with diabetes – such as heart disease – may begin during pre-diabetes^7^.

Globally, an increase in epidemiological trends of prediabetes' prevalence among adolescents has been reported - as in the general population – regardless of the race^8^. The prevalence of Impaired Glucose Tolerance, across age groups, is reported to be 9.7% in Africa; 4.5% in Europe; 7.6% in Middle East and North Africa; 10.7% in North American and Caribbean; 5.4% South and Central American; 3.0% in South East Asia; and 5.4% in Western Pacific^9^. New York State Department of Health noted that among the risk factors for type II DM in children is being African American, Hispanic or Latino American, American Indian, Asian American, or Pacific Islander^10^. However, a review reported highest prevalence among Native Americans and Non-Hispanic Black with prevalence rates of 1.45 and 1.06 per 1000 adolescents respectively; Asian and Pacific Island adolescents have similar prevalence rates of 0.52 and 0.46 per 1000; while the Non-Hispanic White has the lowest prevalence rates 0.18 per 1000 9.

Though the prevalence of prediabetes among children is reaching alarming rates across the globe, the actual global prevalence remains unknown^11^,^12^. A prevalence of DM and prediabetes was reported to 10.83% among children and adolescents in Saudi Arabia 13. In a study among adolescents in Eastern Iran, the FBS in many of the total population (59.6%) was in the upper limit of normal range^14^. In Hungary, 13% of overweight adolescents was at high risk of developing type II DM 15. The prevalence of prediabetes was 5.4% among Emirati overweight/obese children and adolescents^11^. Also, in the District of Abidjan in Cote d'Ivoire, the prevalence of IFG was 14.5 % among children and adolescents^16^.

Despite prediabetes being an emerging public health challenge in sub-Saharan Africa, Nigeria inclusive, there is little research focus on adolescents^17^. Only a few studies have been conducted on its prevalence among Nigerian adolescents. A prevalence of 17% was reported in Port-Harcourt, while 4.0% was reported in Ibadan - using ISPAD criteria^17^,^18^. The need for a similar study to contribute to literature on the prevalence prompted this study.

Social Ecological Model, the theoretical framework for the study, was into three-level to suit the study. The model was used to consider the complex interplay between individual, relationship and community factors. An individual's various traits and identities make up the first level – interpersonal factors. It identifies biological and personal history factors that increase the likelihood of becoming pre-diabetic. Some of these factors are age, gender, financial resources, socio-economic status, race/ethnicity, religious identity, physical health, knowledge, attitudes, behaviour, self-concept, skills, developmental history, genetis, health literacy and personal preferences are some of the many attributes noted at this interval^19^–^21^. The second level of SEM is the interpersonal factors. A person's closest social circle - peers, partners and family members – influences their behaviour^19^. The third level is the community factors. SEM explores the settings, such as schools, workplaces, and neighbourhoods, in which social relationships occur^19^. Hence, the aim of the study was to estimate the prevalence of pre-diabetes, while the specific ones were to the individual, interpersonal and community factors associated with the prevalence of prediabetes among these adolescents.

## Methods

This study utilised a cross-sectional descriptive survey to conduct the study in Osogbo Local Government Area (LGA) – the major LGA in Osogbo metropolis. The target population for this study was secondary school adolescents of the LGA, while study population was the adolescents of the four selected schools. The minimum sample size calculated for this study was 310 participants, using Cochran formula – with adjustment of 20% for non-response and 10% for those the researchers were unable to contact^22^. The estimated sample size was distributed proportionately between public (91.7%) and private (8.3%) schools using enrolment rate^23^. Hence, the minimum sample size for public and private was 285 and 26 respectively. However, Sudman recommended in 1976 that a minimum of 100 participants be recruited in each major group to accommodate a comparative analysis^22^. Hence, the minimum sample size for the study was 385 participants, with sample size of 100 for private school. Eventually, 410 adolescents were recruited using multi-stage sampling technique. However, five was eventually excluded (one for being absent and four for failing to fast during the week of data collection). Hence, 405 adolescents participated in the study.

Inclusion criteria were secondary school adolescents between the ages of 10 and 19 years^24^, who had not been diagnosed with DM and whose parents gave informed consent. Exclusion criteria were adolescents who on the day of study, were absent, ate before coming and/or sick. An interviewer-administered questionnaire, which had eight sections with forty main items, was used. Five sections were developed by the researchers, while three sections were adapted from questionnaire on knowledge of women on gestational diabetes, Godin Leisure-Time Exercise Questionnaire and Adolescent Food Habits Checklist respectively^25^–^27^.

Approval letter and ethical approval certificate were obtained from Osun State Ministries of Education and Health respectively, after which permission was sought from respective school principals. An information sheet/consent form was sent to parents of all adolescents attending the selected schools. Only adolescents whose parents signed the consent form were included in the study. Parents were asked to ensure their children did not take breakfast on the day of the study until they have been tested. For the adolescents, purpose and procedure of the study, as well as the importance of not eating until the test is done were explained to thm on the day prior data collection. After this, adolescents who met the inclusion criteria and gave verbal assent were interviewed. Confidentiality of all data gathered was maintained. To avoid contamination, universal safety precautions were strictly adhered to when carrying out the finger prick test. For ethical reasons, awareness on prediabetes was created among parents, teachers and students. Results of the tests were communicated to the adolescents and their parents by sending Short Message Service (SMS) to their respective parents; and all students with abnormal glucose and Blood pressure (BP) levels were referred to their family physician.

Participants' B/P was first checked, to avoid being stressed, with the aid of Accosson mercury sphygmomanometer - while sitting and well comfortable on chair. After this, the FBG was measured by finger prick test with the aid of Accu-chek active glucometer while still on sit. Weight and height were then be measured while standing on the Generic Height and Weight with no shoes. Body Mass Index (BMI) was categorized according to BMI percentile charts for age and sex by WHO using BMI-for-Age growth charts into underweight (< 5^th^ percentile), normal weight (5^th^ to < 85^th^ percentile), overweight (85^th^ to < 95^th^ percentile) and obesity (≥ 95^th^ percentile). BP was classified with the aid of 2017 American Academy of Pediatrics Guidelines for Childhood Hypertension. The BP percentiles based on age, gender and height was then determined and classified^28^. Meanwhile, FBG result was classified based on the ISPAD criteria. Data were coded and entered into computer with the aid of Statistical Packages for Social Sciences – version 24. Descriptive statistics was used to analyse the main objective. Binomial logistic regression test was used to analyse all the hypotheses, where values of p < 0.05 were considered statistically significant and P < 0.01 were considered highly statistically significant.

## Results

The mean age of the participants was 15.40+2.51; from this, those whose age was 10–15 years were regarded as ‘middle adolescents’ while those 16–19 years were ‘late adolescents’. Hence, 50.6% (n=205) were middle adolescents, while 49.4% (n=200) were late adolescents. Also, 74.6% (n=302) were females, while 25.4% (n=103) were males; 44.9% (n=182) were Christians and 55.1% (n=223) were Muslims; 74.3% (n=301) were from public schools while 25.7% (n=104) were from private schools.

The mean weight of participants was 46.3 + 10.4kg, median height 1.57m, mean BMI 18.7 + 3.1kg/m^2^, mean FBG 88.4 + 9.2mg/dl, mean Systolic BP 102.5 + 12.3 mmHg while the mean Diastolic BP was 66.6 + 11.5mmHg. Furthermore, it was deduced that 20.2% (n=82) of participants were underweight, 73.8% (n=299) had normal BMI, 3.5% (n=14) overweight while 2.5% (n=10) were obese; 88.4% (n=358) had normal BP, 7.9% (n=32) had elevated BP, 3.0% (n=12) had stage I hypertension while 0.7% (n = 3) had stage II hypertension. From figure I, 90.6% (n=367) had normal FGB level, 9.4% (n = 38) had pre-diabetes and none was diabetic. Hence, the prevalence rate was 9.4%. Out of the 38 prediabetic participants, 3 were hypertensive, 5 had elevated BP and 30 had normal BP; while for BMI, 3 was underweight, and 35 had normal BMI (see table I). It is worthy of note that there is positive correlation between the BP and BMI of the participants.

**Figure I:**
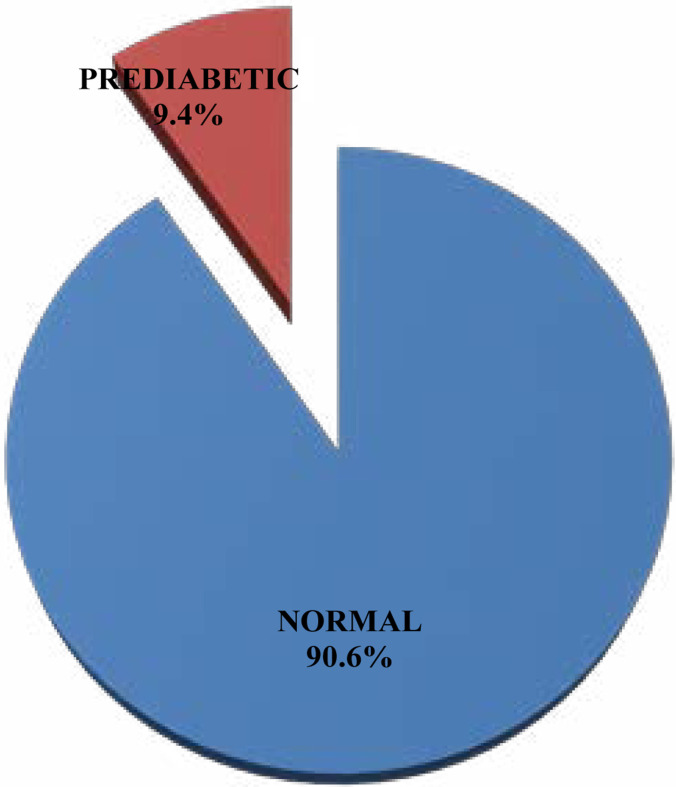
Distribution of pre-diabetes among secondary school adolescents in Osogbo Local Government Area

**Table I:** Crosstabulation of FBG, BP and BMI of participants

BP Category			FBG Category		Total
			Normal	Prediabetic	
Hypertension	BMI category	Overweight/Obese	1	0	1
		Normal	8	3	11
		Underweight	3	0	3
	Total		12	3	15
Elevated BP	BMI category	Overweight/Obese	5	0	5
		Normal	19	5	24
		Underweight	3	0	3
	Total		27	5	32
Normal	BMI category	Overweight/Obese	18	0	18
		Normal	237	27	264
		Underweight	73	3	76
	Total		328	30	358
Total	BMI category	Overweight/Obese	24	0	24
		Normal	264	35	299
		Underweight	79	3	82
	Total		367	38	405

For the first hypothesis, which predicts no statistically significant relationship between the individual factors (socio-demographic variables, lifestyle, physical health, knowledge and genetics) and presence of pre-diabetes, 90.6% of cases were correctly predicted by the model. Among the individual factors tested, only age, religion and family history were statistically significant (see table II). However, BMI was though insignificant, having normal BMI was statistically significant (p = 0.017) when compared to underweight adolescents. BP was also insignificant, but having hypertension was statistically significant when compared to those with normal BP.

**Table II:** Variables in the Equation for Hypothesis One

		B	S.E.	Wald	Df	Sig.	Exp(B)
Step 1	Age	-.232	.081	8.203	1	.004	.793
	Gender(1)	.195	.474	.169	1	.681	1.215
	Religion(1)	-1.487	.498	8.895	1	.003	.226
	Type of school(1)	.498	.474	1.100	1	.294	1.645
	Physical activity	.010	.008	1.665	1	.197	1.010
	Dietary habit	.138	.108	1.646	1	.200	1.148
	BMI Category			5.663	2	.059	
	Overweight/Obese	-17.878	7491.558	.000	1	.998	.000
	Normal BMI	1.692	.711	5.663	1	.017	5.432
	BP Category			4.750	2	.093	
	Hypertension	1.626	.797	4.161	1	.041	5.086
	Elevated BP	.694	.664	1.092	1	.296	2.002
	Knowledge	-.080	.140	.328	1	.567	.923
	Family history (1)	1.463	.605	5.840	1	.016	4.318
	Constant	-1.572	1.483	1.124	1	.289	.208

For the second hypothesis, which predicts no statistically significant relationship between the interpersonal factors (parent's socio-economic status, parent's lifestyle and friend's lifestyle) and presence of pre-diabetes, 91.4% of cases were correctly predicted by the model. Among the interpersonal factors tested, only parents' dietary habit was statistically significant (See table III). However, further analyses on parent's occupation showed higher risk of developing prediabetes is statistically significant in adolescents whose parents were traders/businessmen and public servants when compared those with unemployed parents.

**Table III:** Variables in the Equation for Hypothesis Two

		B	S.E.	Wald	Df	Sig.	Exp(B)
Step 1	Parent occupation			8.268	4	.082	
	Farmer/Artisan	-21.211	11495.452	.000	1	.999	.000
	Trading/Business	-2.552	.894	8.150	1	.004	.078
	Public servant	-2.152	.925	5.415	1	.020	.116
	Private sector worker	-21.229	7381.539	.000	1	.998	.000
	Parent level of education			5.235	3	.155	
	No formal education	1.111	.755	2.164	1	.141	3.036
	Primary education	1.212	.773	2.463	1	.117	3.361
	Secondary education	.806	.432	3.482	1	.062	2.238
	Parent physical activity			2.559	2	.278	
	No activity	-1.715	1.113	2.376	1	.123	.180
	Some activity	-.070	.493	.020	1	.888	.933
	Parent dietary habit	-.665	.238	7.787	1	.005	.514
	Friend physical activity (1)	-.538	.534	1.016	1	.314	.584
	Friend dietary habit (1)	.099	.485	.042	1	.838	1.104
	Constant	1.985	1.261	2.478	1	.115	7.281

For the third hypothesis, which predicts no statistically significant relationship between the community factors (home, school and neighbourhood) and presence of pre-diabetes, 90.6% of cases were correctly predicted by the model. Among the community factors tested, only availability of healthy food in school was statistically significant (see table IV). Further analysis showed that availability of food, fruits and vegetables in school were highly statistically significant when compared with availability of soft drink, snacks and biscuits in school.

**Table IV:** Variables in the Equation for Hypothesis Three

		B	S.E.	Wald	df	Sig.	Exp(B)
Step 1	Home healthy food availability (1)	-.654	.396	2.732	1	.098	.520
	School's sport facilities (1)	.278	.528	.276	1	.599	1.320
	School's safety (1)	-18.784	6718.237	.000	1	.998	.000
	School's healthy food availability			15.599	2	.000	
	Food	1.464	.438	11.178	1	.001	4.322
	Fruits and Vegetables	1.890	.662	8.145	1	.004	6.619
	Neighborhood healthy foods availability			3.455	2	.178	
	Food	-19.462	5966.312	.000	1	.997	.000
	Fruits and Vegetables	-1.224	.658	3.455	1	.063	.294
	Neighborhood safety (1)	-1.193	1.047	1.298	1	.255	.303
	Constant	-1.948	.317	37.683	1	.000	.143

## Discussion

Considering the age distribution of participants with elevated parameters, more overweight participants were middle adolescents, while majority of the obese were late adolescents. This means that as they grow older, overweight adolescents are likely to become obese unless they modify their lifestyle^29^. This trend is similar for the development of hypertension, as there are more middle adolescents with elevated blood pressure and stage I hypertension; but more late adolescents with stage II hypertension. Likewise more middle adolescents were pre-diabetic, compared to their late counterparts. Therefore, there is need to address these disorders at early teen years for effective prevention. Furthermore, considering the gender distribution of participants with elevated BMI, more females were overweight and obese when compared to their male counterparts – such finding was reported in Sokoto state^30^.

This study reported the prevalence rate of prediabetes among the participants to be 9.4%. This implies prediabetes is becoming common in our communities. Urgent steps are therefore needed to be taken to curtail its menace. Such steps include raising awareness of the disease and regular screening exercise among this population - as none of the participants had a previous knowledge of having the disease. This finding is in line with that of previous studies conducted in within and outside Nigeria^13^,^16^–^18^. It also affirms the report of global increasing trend of prediabetes prevalence, with majority unaware of their status^13^,^31^.

In addition, among the individual factors, age, religion and family history were statistically significant in developing prediabetes. That is, participants who are in middle adolescence, Muslim and had family history of DM are at more risk of developing prediabetes than their counterparts in late adolescence, Christian and had family history respectively. Meanwhile, findings from several studies have identified age as significant risk factor for development of pre-diabetes^13^,^18^,^31^,^32^. On family/genetic history of DM, findings from several studies confirm the findings of this study. According to these studies, one of the risk factors for developing pre-diabetes and type II DM in children is having a parent/close relative with type II DM 10,11,15,16,18,32.

However, gender, type of school attended, physical activity, dietary habit and knowledge on diabetes mellitus were not statistically significant. As for gender, the finding was corroborated by that conducted in Cote d'Ivoire, which reported no significant difference in glycemic status of participants in terms of gender^16^. Finding from other studies was not in line with this study, as male gender was identified as significant risk factor for Impaired Fasting Glucose^13^,^14^,^33^. This variation may be as a result of socio-cultural difference from that of Africa, as these studies were conducted in Brazil and Arabian countries. For type of school attended, the finding of this study was not in line with that conducted in Ibadan, where attending private shool was reported as a factor increasing the odds for prediabetes^17^. Similarly on physical activity and dietary habit, the finding was not corroborated by others' findings; several studies reported risk factors for type II DM and prediabetes in children to include lack of physical activity or poor diet^10^,^17^,^33^,^34^. There is therefore need for further study to investigate this variation.

Further findings revealed that those with normal BMI are likely to develop prediabetes while obese/overweight adolescents are not, when compared to those that are underweight. This finding is not in line with many previous studies, which reported overweight/obesity as a risk factor associated with developing prediabetes and DM^10^,^13^,^15^,^17^,^33^,^35^,^36^. However, this finding is in line with that conducted in Port-Harcourt, where no statistically significant difference in the prevalence of prediabetes between normal weight and overweight/obese students was reported^18^. There is need for further study to compare the prevalence of pre-diabetes among underweight, normal, and overweight/obese adolescents, because almost all the previous studies compared between normal and overweight/obese adolescents. Hypertensive adolescents were also reported to likely develop prediabetes when compared to those with normal BP. This finding was corroborated by that of several studies, which reported hypertension as a risk factor for developing of prediabetes and DM^10^,^15^,^17^,^18^,^31^–^33^.

Among the interpersonal factors tested, only parents' dietary habit was statistically significant. That is, the healthier the dietary habit of the parents, the less likely their adolescents developing prediabetes. This finding is in line with that of previous studies^37^,^38^. They explained parents influence youth's eating behaviours through more unfavourable modelling of their parents, and by engaging in practices that affect availability of foods and beverages^38^. More so that parents are responsible for the food their children eat, the rules they follow, and the access they have to resources that promote and hinder positive health behavior^37^. Therefore parents need to be involved when counselling adolescents on their dietary habits for better results. The findings, that parents' physical activity habit was not statistically significant in their children developing prediabetes, was in line with report that even when parents take active role in the amount of physical activity their child participates in, it does not translate to reducing their risk of developing prediabetes^37^. Similarly, physical activity and dietary habit of friend were also not statistically significant in having prediabetes. This finding is not in consensus with the report that Type II DM occurs typically in adolescence, when peer influence predominates^32^. Findings also revealed adolescents whose parents were employed -traders, businessmen/women and public servants -were more likely to develop prediabetes compared to those with unemployed parents. This finding is in consensus with that of previous studies, which reported high family income as significant risk factor for DM and prediabetes^11^,^13^,^31^,^37^.

Lastly, among the community factors, only availability of healthy food in the school was statistically significant. That is, the more availability of food, fruits and vegetables and less availability of soft drink, snacks and biscuits in school, the less likelihood of the adolescents developing prediabetes. This implies schools are important stakeholder when considering healthy dietary habits in adolescents. This finding was in accordance with the report that schools play a critical role in improving the dietary behaviour of adolescents, by creating environments that are supportive of healthy eating and implementing policies and practices^39^. Other factors like availably of healthy food in the home, availability of sport facilities in school, school safety for sport, availability of healthy food in the neighbourhood, neighbourhood safety for exercise were not statistically significant in having pre-diabetes. This finding was not corroborated by previous studies. For instance, it has been documented that increased traffic and fast food locations, as well as decreased recreational space and safe sidewalks – resulting from environmental changes in communities – are affecting children's physical activity, sedentary, and nutrition behaviors^40^. It was also reported that children who were not attending a school considered to be safe had an increased likelihood of being overweight/obese^37^. This variation could be as a result of the study setting, which is a small, developing city, with little or no traffic and limited number of fast food locations.

## Conclusion

Pre-diabetes is becoming a common problem among Nigerian adolescents, like their counterparts worldwide. There are many challenges associated with this; as this is an emerging health issue for this population. There is therefore need for stakeholders at all levels, to face this challenge before it becomes endemic problem in our country.
